# Acute effects of cocaine and cannabis on reversal learning as a function of *COMT* and *DRD2* genotype

**DOI:** 10.1007/s00213-015-4141-5

**Published:** 2015-11-17

**Authors:** Desirée B. Spronk, Marieke E. Van der Schaaf, Roshan Cools, Ellen R. A. De Bruijn, Barbara Franke, Janelle H. P. van Wel, Johannes G. Ramaekers, Robbert J. Verkes

**Affiliations:** Department of Psychiatry, Radboud university medical center, Nijmegen, The Netherlands; Donders Institute for Brain, Cognition and Behaviour, Radboud University, Nijmegen, The Netherlands; Expert Center for Chronic Fatigue, Radboud university medical center, Nijmegen, The Netherlands; Department of Clinical Psychology, Leiden Institute for Brain and Cognition, Leiden University, Leiden, The Netherlands; Department of Human Genetics, Radboud university medical center, Nijmegen, The Netherlands; Department of Neuropsychology and Psychopharmacology, Faculty of Psychology and Neuroscience, Maastricht University, Maastricht, The Netherlands; Pompestichting for Forensic Psychiatry, Nijmegen, The Netherlands; Department of Criminal Law and Criminology, Radboud University, Nijmegen, The Netherlands

**Keywords:** Cocaine, Cannabis, THC, Reversal learning, *COMT* Val108/158Met, *DRD2* Taq1A, Polymorphism, Individual differences, Human

## Abstract

**Rationale:**

Long-term cannabis and cocaine use has been associated with impairments in reversal learning. However, how acute cannabis and cocaine administration affect reversal learning in humans is not known.

**Objective:**

In this study, we aimed to establish the acute effects of administration of cannabis and cocaine on valence-dependent reversal learning as a function of *DRD2* Taq1A (rs1800497) and *COMT* Val108/158Met (rs4680) genotype.

**Methods:**

A double-blind placebo-controlled randomized 3-way crossover design was used. Sixty-one regular poly-drug users completed a deterministic reversal learning task under the influence of cocaine, cannabis, and placebo that enabled assessment of both reward- and punishment-based reversal learning.

**Results:**

Proportion correct on the reversal learning task was increased by cocaine, but decreased by cannabis. Effects of cocaine depended on the *DRD2* genotype, as increases in proportion correct were seen only in the A1 carriers, and not in the A2/A2 homozygotes. *COMT* genotype did not modulate drug-induced effects on reversal learning.

**Conclusions:**

These data indicate that acute administration of cannabis and cocaine has opposite effects on reversal learning. The effects of cocaine, but not cannabis, depend on interindividual genetic differences in the dopamine D2 receptor gene.

**Electronic supplementary material:**

The online version of this article (doi:10.1007/s00213-015-4141-5) contains supplementary material, which is available to authorized users.

## Introduction

Reversal learning is the ability to flexibly adapt behavior in response to changing stimulus–outcome contingencies. It is a cognitive function that is frequently reported to be affected by drug use. Preclinical research has revealed that cannabis and cocaine are associated with impaired reversal learning (Egerton et al. 2005; Sokolic et al. 2011; Wright et al. 2013; McCracken and Grace 2013; Schoenbaum et al. 2004). Furthermore, chronic cocaine use in human addicted individuals has been associated with impaired flexible behavior (Ersche et al. 2008). Impaired reversal learning is also a dimension of impulsivity-related traits. Trait impulsivity, which includes reversal learning, has been related to enhanced drug self-administration levels in rodents (Cervantes et al. 2013; Izquierdo and Jentsch 2012; Dalley et al. 2007). How acute effects of drugs of abuse causally affect reversal learning in humans is yet to be established. Especially for cocaine, acute effects are often fundamentally different from chronic use. Long-term studies often show impairments on cognitive functions, while acute administration most often yields cognitive enhancing effects (Fillmore et al. 2006; Garavan et al. 2008; Spronk et al. 2013, 2015). Here, we examined reversal learning following the acute administration of cannabis and cocaine, the two most commonly used illicit drugs in Europe (EMCDDA 2014). We also investigated drug-induced effects on reversal learning as a function of genetic variants in two common dopaminergic candidate genes.

None of the previous studies on cannabis and cocaine have dissociated between reversal based on unexpected reward versus reversal based on unexpected punishment. This issue is pertinent, because cannabis and cocaine have been argued to act by way of modulating dopamine transmission, which is accompanied by a shift in learning from reward versus punishment (Maia and Frank 2011). Increases in striatal dopamine transmission, as elicited by cocaine (Volkow et al. 1997; Wise 1984), are accompanied by better reward- versus punishment-based reversal learning (Cools et al. 2009; see also Frank et al. 2004; Frank and O’Reilly 2006; Bódi et al. 2009). In agreement, cocaine has been shown to enhance reward-magnitude in rats (Roesch et al. 2007) and thus might cause an overall bias to reward over punishment-related information. This suggests that cocaine might increase the impact of unexpected reward on reversal learning—thus improving reward versus punishment reversal learning. Cannabis has also been associated with increases in striatal dopamine release (Bossong et al. 2009); however, others have not been able to replicate this effect (Stokes et al. 2009; Barkus et al. 2011). Current evidence has also suggested that cannabis reduces sensitivity to external reinforcing stimuli irrespective of their valence (Lane et al. 2002, 2005), which suggests that the valence of the reinforcer does not play a role in shaping learning after reversals, but rather has less of an impact on learning overall.

Individual differences in baseline levels of dopamine synthesis capacity have been shown to be predictive of the effects of dopaminergic drugs on reversal learning (Cools et al. 2009). Accordingly, the effects of cannabis and cocaine might also depend on individual differences in baseline levels of dopamine (de Wit 1998). Thus, we exploited common polymorphisms in dopamine genes to take into account such interindividual differences.

The A1 allele of the *DRD2* Taq1A polymorphism is associated with lower D2 receptor density and hence decreased dopamine D2 receptor signaling (Ritchie and Noble 2003; Jönsson et al. 1999). Moreover, dopamine D2 receptor function—whether or not investigated by means of polymorphisms or pharmacology—has frequently been reported to be associated with individual differences in reversal learning (Lee et al. 2007; Jocham et al. 2009; van der Schaaf et al. 2014) and reinforcement learning in general (Eisenegger et al. 2014). Variation of the *COMT* Val108/158Met is associated with dopamine turnover in the prefrontal cortex. Carriers of the *COMT* Val/Val allele show increased *COMT* enzyme activity and, consequently, decreased dopamine levels in comparison to Met homozygotes (Chen et al. 2004; Tunbridge et al. 2006). Val homozygotes are thought to exhibit the largest cognitive benefit from dopamine-enhancing substances such as *d*-amphetamine and modafinil (Mattay et al. 2003; Bodenmann et al. 2009; but also see Wardle et al. 2013a). In addition to the role of these two genotypes in cognition, both *DRD2* and *COMT* genotypes are implicated in the development of addiction (Blum et al. 1995; Munafò et al. 2007; Tunbridge et al. 2012) and may therefore be involved in modulating the acute effects of drugs of abuse.

In this study, we investigated the acute effects of cannabis and cocaine in healthy regular users of these drugs. All participants received cocaine, placebo, or cannabis in a placebo-controlled double-blind crossover study design. A reversal learning paradigm was employed that is well established to be sensitive to dopaminergic drug manipulations (Cools et al. 2006, 2009; van der Schaaf et al. 2013, 2014).This paradigm enabled us to assess (1) whether cannabis and cocaine alter reward-based vs. punishment-based reversal learning and (2) if drug-induced effects on reversal learning varies as a function of genetic variation in two dopaminergic genes (*DRD2* Taq1A and *COMT* Val108/158Met). In order to investigate the functional selectivity of the effects to reversal learning, we also investigated attention switch task (AST) and forward planning (tower of London: ToL). Functional neuroimaging has most consistently identified the implication of the prefrontal cortex during set-switching (Monsell 2003). Forward planning implicates the prefrontal cortex and striatal dopamine, as evidenced by studies with patients with Parkinson’s disease, characterized primarily by severe striatal dopamine depletion and psychopharmacological studies using drugs that act primarily on striatal dopamine receptors (Newman et al. 2003; Ravizza and Carter 2008; Dagher et al. 2001; Owen et al. 1998; Mehta et al. 2003; van Wel et al. 2013).

Cocaine was predicted to enhance reward relative to punishment-based reversal learning. For cannabis, two hypotheses were deemed possible. First, cannabis might improve reward versus punishment reversal learning as a result of the dopamine-enhancing effects. Second, cannabis might have a valence-independent impairing effect on reversal learning, consistent with prior observations that cannabis reduces sensitivity to external reinforcing stimuli and impairs other executive functions (e.g., Spronk et al. 2015). Furthermore, we hypothesized that these drug effects might vary as a function of individual genetic differences in the *COMT* Val108/158Met and *DRD2* Taq1A polymorphisms.

## Methods

### Subjects

Sixty-four healthy regular (non-addicted) poly-drug users, aged 18–40 years were recruited through advertisements on the Internet, university campuses, and word-of-mouth referrals. A total of 61 subjects remained in the study, because 3 had to be excluded. One withdrew consent after the first testing day, one had a cardiovascular reaction to the blood draw and study discontinuation was decided by the investigators, and one did not adhere to the abstinence instructions as confirmed by high baseline cannabinoid levels for each testing day. All subjects reported regular cannabis use (i.e., two or more times per week) and occasional cocaine use (i.e., more than five times in the previous year). They had to be in good physical health and be of normal weight (body mass index 18–28). Subjects who used other psychotropic medication, reported excessive drinking (>20 standard drinks per week) or smoking (>20 cigarettes per day) were excluded. Further exclusion criteria were alcohol or substance dependence or history of psychiatric or neurological disorders as assessed during a clinical interview (M.I.N.I. plus; Sheehan et al. 1998), pregnancy or lactation, and cardiovascular abnormalities as measured by ECG and hypertension. Not all subjects completed all tasks in all three drug conditions. Of the 61, 3 subjects did not complete the attention switch task and 5 did not complete the tower of London in the cannabis drug condition (because of adverse events). Eight additional datasets of the tower of London could not be analyzed due to experimenter error (1 cocaine, 1 placebo) or to non-compliance or failure to understand the task by the subject as reflected in an overall performance of less than 60 % correct (3 cannabis, 2 placebo, 1 cocaine). There were 14 missing datasets for the reversal learning due to adverse events or lack of motivation (13 cannabis, 1 cocaine). Data for 4 additional datasets were excluded due to poor behavioral performance (overall average accuracy <60 %; 2 cannabis, 1 placebo, 1 cocaine).

The study was part of a multicenter trial, but the current results were collected at one study site. The reversal learning task, attention switch task, and the tower of London were administered as part of a larger cognitive test battery (see Dutch Trial Register, trial number NTR2127; results will be published elsewhere). The study was conducted according to the code of ethics on human experimentation established by the Declaration of Helsinki (1964, amended in Seoul 2008), and was approved by the Medical Ethics Committee of Maastricht University and the regional ethics committee for the University Medical Center of the Radboud University. A permit for obtaining, storing, and administering cocaine and cannabis was obtained from the Dutch Drug Enforcement Administration.

### Procedure

Prior to starting the testing days, all participants were invited for a screening and practice day where they gave informed consent and received a medical examination including assessment of blood and urine samples for standard chemistry and hematology, electrocardiogram (ECG), and interview of medical and drug use history. All subjects completed shortened versions of all cognitive paradigms in a practice session.

**Fig. 1:**
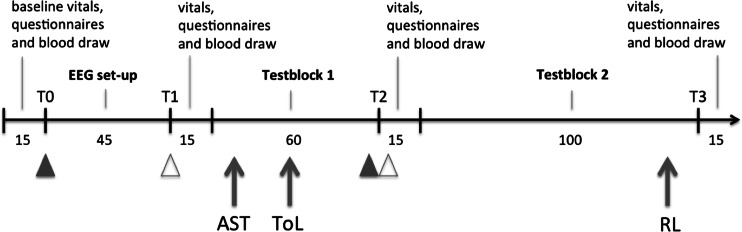
Timeline (in minutes) of the course of a testing day. The *black triangles* represent the moment of cocaine (or placebo) capsule administration and the *gray triangles* represent the moment of cannabis (or placebo) vapor administration. *AST* attention switch task, *ToL* tower of London, *RL* reversal learning

After study inclusion, subjects completed a series of cognitive tests on three separate testing days that were separated by at least a week. Subjects were asked to abstain from caffeine and nicotine on the testing day and from cannabis and alcohol at least 24 h prior to each testing day. Figure 1 shows the timeline of procedures on a testing day. The testing day started with a light breakfast (non-caffeinated tea or water, up to four sandwiches) and performance of a urine drug screen, pregnancy test (women only), and alcohol breath analyzer. This was followed by pre-drug (baseline) vital sign recordings, subjective questionnaires, and blood draws (see supplementary material 1 for drug metabolites and see van Wel et al. 2015, for questionnaire data). Subjects received a capsule containing either 300 mg cocaine HCl or placebo orally (T0), and 45 min later, subjects inhaled 300 μg/kg cannabis or placebo (T1); because the experiment was double-blind, therefore, all participants had to take a capsule and complete inhalation. The delay of 45 min was based on prior observations that the capsule needs about 45 min before plasma concentrations start to increase (Fillmore et al. 2002; and for a review Bigelow and Walsh 1998). Conversely, cannabis was anticipated to be absorbed immediately (Grotenhermen 2003). After T1, the first block of behavioral tasks was assessed (block 1) during which the attention switch task and tower of London were completed. About 1 h after T1, a second booster dose of cocaine (150 mg) or placebo followed by a second dose of cannabis 150 μg/kg or placebo was given (T2). Next, the second block (block 2) of behavioral tasks was administered. The reversal learning task was at the end of this second block. Vital sign recordings, subjective questionnaires, and blood draws were obtained 5 min after drug administration (T1 and T2) and at the end of the testing day. An extra vital sign recording was performed before T2 to assess the safety of administering the potential booster.

Of the 59 subjects who completed the reversal learning task in the cocaine condition, 16 did not receive the second booster capsule. Five subjects did not receive a second cocaine dosage, because the decision to include a second booster dosage was made after the start of the study and approval for this amendment had to be awaited. Eleven subjects’ vital signs exceeded limits for safe administration of the booster. Supplementary analyses of the effects of cocaine after exclusion of these 16 subjects revealed a similar pattern of the results. Of the 46 subjects who completed the reversal learning task in the cannabis condition, 6 did not receive a second administration (3 refused, 3 exceeded vital sign limits).

### Design, study drugs, and administration

This study used a double-blind, double-dummy, placebo-controlled, three-way crossover design. The three possible conditions were as follows: (1) cocaine (placebo cannabis vapor/cocaine capsules), (2) cannabis (cannabis vapors/placebo cocaine capsules), and (3) placebo (placebo cannabis vapors/placebo cocaine capsules). Cannabis was obtained from flowers of *Cannabis sativa,* grown according to good manufacturing practice (GMP)-compliant procedures (FarmalyseBV, Zaandam, The Netherlands). A herbal mixture containing hemp flowers was used as placebo for cannabis. Cannabis was administered in two subsequent dosages that were tailored to each individual’s weight (T1 = 300 μg/kg, T2 = 150 μg/kg). Cannabis and cannabis placebo were vaporized at a temperature of 225 °C by means of a Volcano® vaporizer (Storz-Bickel GmbH, Tüttlingen, Germany) 5 min before administration. The vapor was stored in a polythene bag equipped with a valved mouthpiece. Subjects were instructed to inhale deeply, to hold their breath for 10 s after each inhalation and to take as much time as needed to empty the bag in order to minimize the occurrence of adverse events. Cocaine HCl and matching placebo cocaine were encapsulated in white opaque capsules. The placebo capsules contained only filling material of equivalent weight. The cocaine HCl and placebo cocaine were purchased from Mallinckrodt Pharmaceuticals, St. Louis, MO, USA, and encapsulated and tested by Basic Pharma Geleen, The Netherlands, according to Good Manufacturing Practices. Two subsequent dosages of cocaine capsules (T0 = 300 mg, T2 = 150 mg) or placebo capsules were administered. The capsules were taken orally with 150 ml of water. The sequence of the drug conditions was counterbalanced.

### Genetics

Blood samples were obtained by venipuncture, and DNA was isolated using the following standard protocols. Molecular analyses were performed in a certified laboratory at the Department of Human Genetics, Radboud University Medical Center, the Netherlands. The *DRD2* rs1800497 and *COMT* rs4680 polymorphisms were genotyped using TaqMan-based analysis. Genotyping was performed in a volume of 10 μl containing 10 ng of genomic DNA. For *DRD2*, 5 μl of TaqManMastermix (2x; Applied Biosystems, Nieuwerkerk aan de IJssel, the Netherlands), 0.125 μl of TaqMan assay (TaqMan assay: C_7486676_10, reporter 1, VIC-A-allele, reverse assay; Applied Biosystems), and 3.875 μl of water were added. For *COMT*, 5 μl of ABgene Mastermix (2x, Applied Biosystems), 0.125 μl of TaqMan assay (TaqMan assay: C_25746809_50, reporter 1, VIC-A-allele; Applied Biosystems), and 3.875 μl of water were added. Amplification was performed on a commercially available system (7500 Fast Real-Time PCR, Applied Biosystems), starting with 15 min at 95 °C, followed by 50 cycles of 15 s at 95 °C and 1 min at 60 °C. Genotypes were scored using the algorithm and software supplied by the manufacturer (Applied Biosystems). To investigate the random genotyping error rate in the two assays, the laboratory included 5 % duplicate DNA samples, which showed 100 % consistency in genotype. There are three genotypes of the dopamine *DRD2* Taq1A gene; the A2/A2 variant, the A1/A2 variant, and the A1/A1 variant. The A1/A1 and A1/A2 variants were grouped together and named the “A1 carriers,” because the prevalence of the A1/A1 variant is very low. There are three variants for the COMT gene; the Val/Val variant, the Val/Met variant, and the Met/Met variant. The *COMT* genotype could not be determined for two subjects. The observed distribution of both genotypes was in agreement with expected values according to the Hardy–Weinberg equilibrium (*p*_DRD2_ = 0.61; *p*_COMT_ = 0.13).

### Tasks

#### Reversal learning

**Fig 2:**
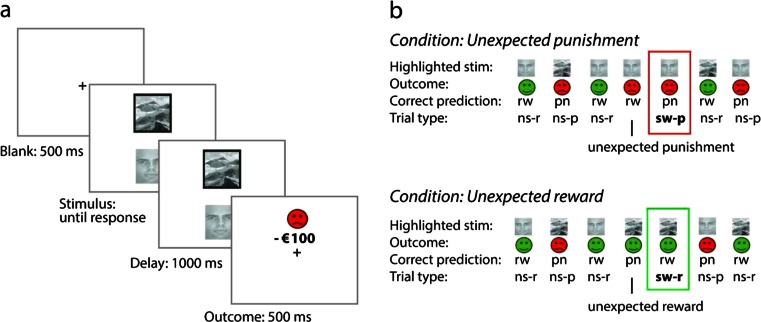
The deterministic reversal learning task. **a** An example of a punishment trial. On each trial, subjects are presented with a face and a landscape. One of the images is surrounded by a *black border*. The subject had to *predict* whether the surrounded stimulus was followed by a reward or by a punishment outcome by pressing the associated “reward” or “punishment” button on a keyboard. After the response had been made, the subject saw the actual outcome. **b** Example of a trial sequence for the unexpected punishment and unexpected reward condition. The reversals are indicated by an unexpected reward or unexpected punishment trial. *rw* reward prediction, *pn* punishment prediction, *ns-r* non-switch reward trial, *ns-p* non-switch punishment trial, *sw-r* reversal trial after an unexpected reward, *sw-p* reversal trial after an unexpected punishment

A deterministic reversal learning paradigm was used (see Fig. 2 and Cools et al. 2006), in which subjects were simultaneously presented with a face and scene picture on a trial by trial basis (location randomized). One of these stimuli was associated with reward and the other with punishment. Subjects were instructed to learn these deterministic stimulus–outcome associations by predicting the outcome of the stimulus that was highlighted by a black border. Outcome predictions were made by pressing either a red (for punishment) or green (for reward) button with the index finger of the left and the right hand (counterbalanced between subjects). Note that the computer selected which stimulus was highlighted and that the outcome was always independent of the subject’s actual response. One second after the button press, the outcome was presented for 500 ms at the location of the stimulus. Reward consisted of a green smiley with a “+€100” sign. Punishment consisted of a red angry smiley and a “−€100” sign. Subjects did not get any monetary rewards. The task was self-paced and no response deadlines were employed. After 4–6 consecutive correct predictions, the stimulus–outcome contingency reversed. This was signaled by either an unexpected reward, presented after the previously punished stimulus was highlighted, or an unexpected punishment, presented after the previously rewarded stimulus was highlighted. After unexpected outcomes, the same stimulus was highlighted again, such that behavioral and cognitive requirements were matched between valence conditions.

Each participant performed four experimental blocks that contained 120 trials: two blocks in which reversals were signaled by unexpected rewards (reward valence) and two blocks in which reversals were signaled by unexpected punishment (punishment valence). Each block consisted of one acquisition stage until the first reversal and a variable number of reversal stages, depending on the participant’s accuracy. Accuracy on the trials directly following these unexpected outcomes (switch trials) represents how well subjects updated their stimulus–outcome associations. The remainder of the trials consisted of non-switch trials in which subjects simply had to predict if the trial was associated with reward (non-switch reward) or punishment (non-switch punishment). Thus, in total, there were three trial types (switch, non-switch reward, non-switch punishment) across two valence levels (reward, punishment). Proportion correct is the main outcome measure in this task. On the “screening and practice day” subjects performed two practice blocks to familiarize them with the paradigm.

#### Attention switch task

The experimental attention switch task (AST) was used to measure the subject’s ability to frequently switch attention between different task instructions (Markus and Jonkman 2007). This task is an adjusted version of the task reported by Kramer et al. (2001). The AST consists of two non-switch blocks and one switch block. During the task, four stimulus types were randomly presented on the screen (1, 3, 111, or 333). During the first non-switch block, the subject was asked to respond to the question “what number?” by pressing a left button when the cue “1” or “111” appeared and a right button when “3” or “333” appeared. In the second non-switch block, the question “how many?” had to be answered by pressing the left button when the cue “1” or “3” appeared and the right button when the cue “111” or “333” appeared. In the switch block, the “what number?” and “how many?” trials were randomly intermixed. The non-switch blocks included 40 randomly presented trials each, consisting of 10 stimuli of each stimulus type. The switch block included 80 randomly presented trials and 20 stimuli of each stimulus type. The questions were presented for 400 ms, after which a stimulus was presented for 800 ms. After this, a 2000-ms response interval occurred before the next instruction appeared. The dependent variables were mean proportion correct and reaction time.

#### Tower of London

The tower of London task provides a measure of forward planning (Shallice 1982). The original version of the Tower of London consists of three colored balls, which must be arranged on three sticks to match the target configuration on a picture while only one ball can be moved at a time. The present version consisted of computer-generated images of begin- and end-arrangements of the balls. The subjects were asked to decide as quickly as possible, by pushing a coded button, whether the end arrangement could be accomplished in 2, 3, 4, 5, or 6 steps from the begin arrangement (Veale et al. 1996). The complexity of the task was dependent on the minimal number of steps in which the rearrangement could be achieved. To avoid guessing, only the trials of two to five steps are analyzed. Proportion of correct decisions and mean reaction time per step were the main outcome measures.

### Data analysis

Potential genotype differences in gender, age, and education were analyzed through Pearson’s chi-square test or univariate ANOVA, as appropriate. To assess drug effects on reversal learning, a linear-mixed model (LMM) was constructed with subject as a random factor and drugs (cocaine, placebo, cannabis); valence (reward, punishment); and trial type (switch, non-switch reward, non-switch punishment) as fixed factors in each of the analyses. To assess whether drug effects on reversal learning depended on individual differences in *COMT* or *DRD2* genotype, an additional LMM was performed with *COMT* (Val/Val, Val/Met, Met/Met) and *DRD2* (A2/A2, A1 carriers), as two additional fixed factors. To measure drug effects on AST and ToL performance, LMMs were constructed with subject as a random factor and drugs (cocaine, placebo, cannabis); block (switch, non-switch, for AST); or step (steps 2, 3, 4, and 5, for ToL) as fixed factors. All individual datasets which involved more than 40 % *overall* error rate were excluded as performance was considered to be at chance level, signaling non-compliance or failure to understand the instructions. As is appropriate for proportional data, where the variance is proportional to the mean, we applied arcsine transformation (2*x*(arcsine(√*x*)) on all analyses on proportion correct (Howell 1997). We present and plot raw data in tables and figures for illustrative purposes. A significant interaction effect was followed by drug–placebo contrasts to establish the separate effects of cannabis and cocaine and their interaction with genotype. Mixed model analysis of variance rather than ANOVA was chosen because subjects for whom data were unavailable for one or two of the three drug conditions could be included in the analysis by assuming values were missing at random.

## Results

### Demographics

Table 1 shows the subject characteristics for gender, age, education and drug use history stratified for genotype. There was a significant *DRD2* genotype effect on occasions of cocaine use in the past year. The A1 carriers showed a higher prevalence of cocaine use compared with the A2/A2 homozygotes. However, the A1 carrier group contained one outlier. There was no significant *DRD2* genotype effect on occasions of cocaine use when this subject was removed from the analyses (*p* = 0.17). There were no other significant genotype effects.

**Table 1:** Demographics of subjects stratified per genotype, values are mean ± SD

	*DRD2 genotype*			*COMT genotype*			
Measure	*A2/A2*	*A1 carriers*	*p* value	*MetMet*	*ValMet*	*ValVal*	*p* value
*N*	44	17		23	23	13	
Gender (M/F)	36/8	13/4	0.64	16/7	19/4	13/0	0.08
Age (years)	22.1 ± 3.8	23.8 ± 5.3	0.17	23.1 ± 4.8	22.1 ± 3.0	23.1 ± 5.4	0.71
Years of education^a^	14.2 ± 2.2	15.0 ± 1.8	0.20	14.6 ± 2.2	14.6 ± 1.9	14.1 ± 2.5	0.80
Occasions of cocaine use (occasions per last year)^b^	8.9 ± 4.7	15.2 ± 18.1	0.04*	9.2 ± 4.7	9.5 ± 6.3	16.0 ± 20.0	0.13
Estimated frequency of cannabis use (J/W)	5.5 ± 4.9	8.0 ± 5.3	0.078	6.6 ± 4.8	6.4 ± 5.9	5.5 ± 4.7	0.83

^a^Data is missing for one subject

^b^When one outlying participant was removed, the DRD2 genotype effect on occasions of cocaine use was no longer significant (*p* = 0.17)

### Reversal learning

The average proportions of correct responses are shown in Table 2. Mixed model analysis revealed a significant main effect of drugs (*F*_2,929.565_ = 76.2, *p* < 0.001), a significant effect of valence (*F*_1,911.998_ = 5.0, *p* = 0.026), and a significant trial type × valence interaction (*F*_2, 911.998_ = 67.20, *p* = 0.001). There was no trial type × drugs interaction (*F*_2, 911.998_ = 0.0.68, *p* = 0.61), suggesting that drug effects were the same for switch and non-switch trials. Moreover, in contrast to our hypothesis, there was no drugs × valence interaction, indicating that drug effects did not vary as a function of the valence of the outcome that signaled the need for reversal. Furthermore, there was also no three-way trial type × valence × drugs interaction (*p*’s > 0.99). The main effect of drugs was due to a higher accuracy after cocaine compared with placebo (M = 0.94 vs. 0.91 *p* < 0.001), but lower accuracy after cannabis compared with placebo (M = 0.87 vs. 0.90, *p* < 0.001). Thus, cocaine improved, while cannabis impaired performance on the reversal learning task in a non-valence specific manner and the effects extended to the non-switch trials.

**Table 2:** Behavioral data on the reversal learning task (mean proportion correct ± SD)

		Cocaine		Placebo		Cannabis	
		A2/A2	A1 car	A2/A2	A1 car	A2/A2	A1 car
		(*N* = 42)	(*N* = 17)	(*N* = 42)	(*N* = 17)	(*N* = 33)	(*N* = 13)
Unexpected reward	Switch	0.91 ± 0.091	0.92 ± 0.087	0.88 ± 0.11	0.84 ± 0.10	0.83 ± 0.15	0.77 ± 0.19
	Non-switch reward	0.95 ± 0.061	0.95 ± 0.039	0.92 ± 0.074	0.91 ± 0.038	0.90 ± 0.080	0.85 ± 0.12
	Non-switch punishment	0.95 ± 0.049	0.94 ± 0.070	0.92 ± 0.089	0.90 ± 0.060	0.91 ± 0.059	0.86 ± 0.094
Unexpected Punishment	Switch	0.96 ± 0.045	0.95 ± 0.051	0.92 ± 0.088	0.87 ± 0.11	0.85 ± 0.17	0.84 ± 0.14
	Non-switch reward	0.95 ± 0.050	0.95 ± 0.034	0.93 ± 0.044	0.91 ± 0.056	0.89 ± 0.079	0.86 ± 0.10
	Non-switch punishment	0. 95 ± 0.051	0.96 ± 0.044	0.93 ± 0.051	0.90 ± 0.054	0.90 ± 0.069	0.88 ± 0.047

Post-hoc analyses of the two-way interaction between valence and trial types showed a significant effect of valence for switch trials, i.e., across all drug sessions subjects performed better when unexpected reversal was signaled by punishment rather than reward (M = 0.90 vs. 0.86, *p* < 0.001). No differences were observed between reward and punishment on the two types of non-switch trials (*p* > 0.75).

### Genetic effects on reversal learning

**Fig. 3:**
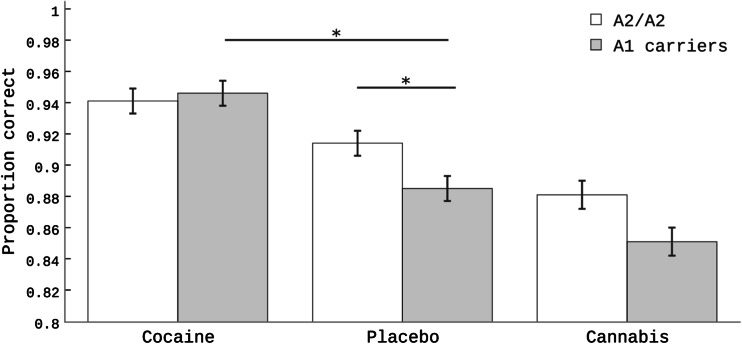
Proportion correct for the cocaine, placebo, and cannabis conditions as a function of *DRD2* Taq1A genotype collapsed across the different trial types (mean ± SEM)

In addition, we assessed whether reversal learning performance was modulated by *DRD2* or *COMT* genotype. The proportion of correct responses as a function of drug, trial type, and *DRD2* genotype (A2/A2 and A1 carriers) are presented in Fig. 3. There was a significant *DRD2* × drugs interaction (*F*_2,841.511_ = 5.33, *p* = 0.005). Follow-up analyses investigating this *DRD2* × drugs interaction for only the cocaine and placebo datasets demonstrated that the cocaine-induced improvement was greater in the A1 carriers compared with the A2/A2 group (*F*_1,589.251_ = 11.9, *p* = 0.001). The *DRD2* × drugs interaction analysis for cannabis and placebo conditions revealed no significant effect (*F*_1,546.422_ = 0.77, *p* = 0.38), suggesting that the effects of cannabis did not differ between A1 carriers and the A2/A2 group. There were no other significant interactions with *DRD2* genotype (all *p*’s > 0.52). There was no significant main effect for *COMT* genotype (*p* = 0.08), neither were there any significant interactions between *COMT* and any of the other factors (all *p*’s > 0.34).

### Attention switch task

The average proportion of correct responses and reaction times for the attention switch and the tower of London tasks are shown in Table 3. Analysis revealed the expected switch effect as indicated by a decreased proportion of correct in the switch compared with the non-switch block (0.97 vs. 0.94; *F*_*1*, 294.289_ = 117.9 *p* < 0.001)*.* There was also a main effect of drugs on proportion correct (*F*_2, 295.572_ = 16.9, *p* < 0.001). Pairwise comparisons revealed that subjects made more errors under the influence of cannabis compared with placebo (0.94 vs. 0.96, *p* < 0.001). There were no differences between the cocaine and placebo condition (0.96 vs. 0.96, *p* = 0.26). The switch × drugs interaction was not significant (*F*_2, 294.289_ = 2.36, *p* = 0.097), suggesting that the switch effect on proportion correct was not different across the three drugs conditions. In addition, in terms of reaction times, there was also a main effect of switch (*F*_1, 294.047_ = 241.9, *p* < 0.001) and of drugs (*F*_2, 294.863_ = 13.3, *p* < 0.001). Subjects took longer to respond to trials in the switch compared with the non-switch block (652 vs. 485 ms). The significant main effect of drugs was due to overall longer reaction times in the cannabis compared with placebo condition (603 vs 568 ms, *p* = 0.031) and faster reaction times in the cocaine compared with the placebo condition (534 vs 568 ms, *p* = 0.028). There was no significant switch × drugs interaction (*F*_2, 294.047_ = 0.036, *p* = 0.96), suggesting that the switch effect on reaction time was not different between the drugs.

**Table 3:** Behavioral data on attention switch task (AST) and tower of London (ToL) after placebo, cocaine, and cannabis (mean ± SD)

		Placebo	Cocaine	Cannabis
Attention switch task				
Non-switch	RT (ms)	483 ± 91	452 ± 67	517 ± 142
	proportion correct	0.98 ± 0.026	0.98 ± 0.024	0.96 ± 0.036
Switch	RT (ms)	654 ± 198	617 ± 148	682 ± 201
	proportion correct	0.95 ± 0.050	0.95 ± 0.036	0.92 ± 0.049
Tower of London				
2 steps	RT (s)	4962 ± 1404	4910 ± 1690	6161 ± 2625
	proportion correct	0.94 ± 0.088	0.95 ± 0.090	0.92 ± 0.12
3 steps	RT (s)	6218 ± 1870	6292 ± 2513	7377 ± 2740
	proportion correct	0.95 ± 0.071	0.95 ± 0.068	0.92 ± 0.093
4 steps	RT (s)	9449 ± 3452	9144 ± 3184	10,914 ± 3909
	proportion correct	0.88 ± 0.10	0.85 ± 0.11	0.82 ± 0.15
5 steps	RT (s)	15,337 ± 4839	15,278 ± 4988	15,760 ± 5904
	proportion correct	0.76 ± 0.18	0.79 ± 0.17	0.69 ± 0.17

### Tower of London

The analyses on the reaction times in the ToL showed the expected decrease in proportion correct with increasing number of steps (*F*_3,607.373_ = 104.3, *p* < 0.001). With the exception of steps 2 and 3, significantly fewer correct responses were made with each subsequent step (all *p*’s < 0.001). There was also a significant effect of drugs (*F*_2,619.725_ = 12.1, *p* < 0.001). Pairwise comparisons revealed that the effect of drugs was due to an overall lower proportion correct after cannabis compared with placebo (*p* < 0.001), while there were no differences between placebo and cocaine (*p* = 1.0). The drugs × steps interaction on proportion correct was not significant (*F*_6*,*619.132_ = 1.0, *p* = 0.42). Likewise, there was a significant main effect of steps on reaction time (*F*_3*,*608.498_ = 560.2, *p* < 0.001), indicating that reaction time increased with each subsequent step (all *p*’s < 0.001). In addition, a main effect of drugs (*F*_2*,*612.612_ = 15.7, *p* < 0.001) indicated that reaction times were longer after cannabis compared with placebo (*p* < 0.001), while there were no reaction time differences between cocaine and placebo (*p* = 1.0). There was no interaction between steps × drugs (*F*_6,608.498_ = 0.76, *p* = 0.61).

## Discussion

The main findings of this study are that (1) cocaine and cannabis exerted opposite effects on reversal learning that are non-specific with respect to valence. Cocaine increased proportion correct whereas cannabis decreased proportion correct. These effects were observed across all trials: switch and non-switch; (2) drug effects on reversal learning did not vary with the valence of the outcome (reward vs. punishment) signaling the need for reversal; and (3) the *DRD2* Taq1A genotype differentially modulated the effects of cocaine, but not cannabis. Specifically, the results suggested that cocaine improved performance to a greater degree in the A1 carriers compared with the A2/A2 genotype group. The *COMT* Val108/158Met did not affect reversal learning.

Cocaine induced a larger improvement in the *DRD2* A1 allele carriers compared with the A2/A2 homozygotes. Furthermore, this was irrespective of switch or non-switch trials, suggesting that this effect reflects modulation of reinforcement learning rather than reversal learning specifically. The finding that cocaine had greater beneficial effects in subjects with genetically determined lower dopamine D2 receptor density concurs with prior pharmacogenetic studies also showing greater effects of dopaminergic drugs in such subjects (Cohen et al. 2007; Kirsch et al. 2006; Pearson-Fuhrhop et al. 2013; Kwak et al. 2013). For example, in the work by Cohen et al. (2007) it was shown that administration of the dopamine D2 receptor agonist cabergoline resulted in stronger task-related neural activation in the A1 carriers compared with the A2/A2 genotype group. The results also accord with a recent pharmacological PET study in healthy individuals (Ersche et al. 2011), which demonstrated that the beneficial effects of the dopamine receptor agonist pramipexole on reversal learning depended on baseline levels of dopamine D_2/3_ receptor availability in the striatum (Ersche et al. 2011). Although cocaine is pharmacologically different from the dopamine precursor l-dopa and the dopamine receptor agonists investigated in the aforementioned studies (Cohen et al. 2007; Kirsch et al. 2006; Pearson-Fuhrhop et al. 2013; Kwak et al. 2013), these studies generally concur to suggest that the degree of cognitive benefit after increasing dopamine transmission is predicted by baseline dopamine D2 receptor availability.

The *DRD2* Taq1A polymorphism did not explain interindividual differences in the effects of cannabis. To the authors’ knowledge there are currently no published cannabis drug studies that investigate the interaction with the *DRD2* Taq1A genotype. Cannabis has a unique and complicated pharmacological profile, involving many neurotransmission systems such as dopamine, GABA, and acetylcholine (Bossong et al. 2009; Stokes et al. 2009; Barkus et al. 2011; Wilson and Nicoll 2002). It is likely that cannabis’ effects on cognition are predominantly mediated by neurotransmitters other than dopamine. More extensive future studies should address the pharmacogenetic effects of cannabis on cognition.

Whether individual differences in acute drug effects on reversal learning could provide information with relevance for drug-using individuals is an intriguing question. Our results showed that the A1 carriers showed the largest cognitive benefits after cocaine. Assuming that cognitive benefits from a drug are a direct motivational reinforcer for use, cocaine’s beneficial effect on reversal learning could further reveal a potential mechanism by which the A1 carriers are more vulnerable for cocaine use. The higher benefit from cocaine for A1 carriers perhaps means that the reinforcing effects are larger for this group. This fits well with studies showing that lowered expression of D2 receptors is associated with stronger pleasurable responses to stimulants (Volkow et al. 1999; Spellicy et al. 2014) and research showing that the A1 allele of the DRD2 Taq1A genotype is associated with greater propensity for drug use and addiction (Persico et al. 1996; Bühler et al. 2015).

In contrast to the *DRD2* Taq1A gene, the *COMT* Val108/158Met genotype did not interact with drug effects. This lack of significant association might be due to a lack of statistical power, which is a common problem in gene-cognition studies. While an effect of *COMT* on cognition has been demonstrated many times (Tunbridge et al. 2006), it can often not be replicated (Barnett et al. 2008; Wardle et al. 2013b). This includes failures to replicate seminal pharmacogenetic findings on which our hypotheses were based (Mattay et al. 2003). The current results thus suggest that better powered studies are needed to establish the role of the *COMT* gene in cognition in general and in combination with pharmacological substances.

Irrespective of the *DRD2* genotype interaction with drugs, it is notable that the impaired learning in the *DRD2* A1 allele carriers compared with the A2/A2 group agrees with a number of studies (Jocham et al. 2009; Klein et al. 2007; Richter et al. 2014). Specifically, these studies indicated that carriers of the gene variant associated with presumably lower D2 receptor density show impaired performance on reinforcement learning (Klein et al. 2007; Frank et al. 2007), as well as impaired avoidance learning (Richter et al. 2014). This also concurs with prior observations from pharmacological work showing that reversal learning performance depends on the degree of dopamine D2 receptor availability (van der Schaaf et al. 2013; Groman et al. 2011). However, our finding of impaired learning in the A1 allele carriers compared with the A2/A2 was irrespective of valence. This contrasts with studies that have linked the *DRD2* polymorphisms to learning from punishment or avoidance learning specifically (Frank and Hutchison 2009; Klein et al. 2007; Frank et al. 2007). However, we note that the paradigms used in the various studies are very different: where we employed a deterministic paradigm in which the outcome was dependent on the stimulus, previous work commonly used a probabilistic reversal learning paradigm in which the outcome was dependent on the response.

Cocaine is a powerful stimulant and the observed improvement on the reversal learning task is in the same direction as the effects of other stimulant drugs such as amphetamine and modafinil on learning tasks in humans (Pessiglione et al. 2006; Wickens 1990; Pringle et al. 2013). Stimulant drugs share comparable pharmacological properties such as increasing dopamine and noradrenaline levels in the brain and enhancement of arousal (Kuczenski and Segal 1994; Berridge 2006). These results support the interpretation that cocaine facilitates overall learning due to its stimulant properties. By contrast, the cocaine-induced performance improvement contrasts with previous observations in rodents and humans showing that prior *chronic* use of cocaine is associated with impaired reversal learning (Ersche et al. 2008; Jentsch et al. 2002; Schoenbaum et al. 2004; Calu et al. 2007; McCracken and Grace 2013). As such, the present results concur with previous findings showing that acute effects of cocaine can be opposite to the chronic effects of cocaine (reviewed in Spronk et al. 2013). Moreover, this cocaine-induced improvement in learning was restricted to the reversal learning task and did not extend to the other tasks. Performance on the attentional switch task and tower of London were unaffected by cocaine, although reaction times on the AST were faster after cocaine. Other studies on stimulant drugs have also failed to show effects on forward planning and switching of attention (van Wel et al. 2013; Hermens et al. 2007; Linssen et al. 2012; but also see Elliott et al. 1997; Rogers et al. 1999). This suggests that cocaine enhances the more cognitive demanding process of reversal learning, but leaves basal functions such as attention switching and forward planning intact.

Additionally, we observed that valence differentially affected learning. Participants seemed to learn better after punishment rather than reward predicting reversal. Most relevant to the current investigation, drugs did not differentially affect the degree of learning from reward versus punishment, but instead only revealed a general improvement on all trial types. For cocaine, the results contrasted with our hypothesis in which we expected relative enhanced improvement in reward versus punishment learning. These results are in striking contrast with previous findings showing opposite effects of dopaminergic agents on reward and punishment learning (Cools et al. 2006; Frank and O’Reilly 2006; Cools et al. 2009; van der Schaaf et al. 2013) even when using the same task (Cools et al. 2006, van der Schaaf et al. 2013). On the other hand, they seem to concur with recent pharmacological studies showing unidirectional effects on both reward and punishment learning (Pessiglione et al. 2006, Jocham et al. 2014). There are several reasons that may explain these contrasting results.

One possible explanation may relate to the instrumental and Pavlovian learning components in our reversal learning task (Van der Schaaf et al. 2013). Subjects could improve on the task by detecting whether the outcome, which solely depends on the stimulus and not on subjects’ actions, is better or worse than expected. This process depends on learned (Pavlovian) stimulus–outcome associations and is measured by the accuracy on the trials directly after a reversal signaled by unexpected rewards and unexpected punishments. Alternatively, subjects could improve on the task by detecting whether their action was correct or incorrect by means of a match or mismatch between their prediction (action) and the outcome. This process depends on instrumental (action-outcome) associations and may be reflected by general accuracy on all trials (irrespective of outcome valence or reversal). Indeed, in previous studies, an instrumental learning task (without reversals) was used in which improvement in both reward-approach as well as punishment-avoid learning was observed (Pessiglione et al. 2006; Jocham et al. 2014). Taken together, our findings that cocaine improved general accuracy might reflect improvements on the instrumental component of our task, which could have overshadowed the (expected) valence-dependent effects.

Alternatively, the lack of valence-specific effects after cocaine may imply enhanced saliency of both reward and punishment signals. Dopamine has been shown to code information related to salient events (Bromberg-Martin et al. 2010; Horvitz 2000) and can predict both reward- and punishment-related information (Matsumoto and Hikosaka 2009; Brischoux et al. 2009). Accordingly, enhanced motivational salience due to elevated dopamine levels could thus have led to equally improved learning from both reward and punishment signals. Third, cocaine is different from the (mostly) agonists and antagonists that were administered in previous work and might therefore exert very different effects. Cocaine does not only enhance dopaminergic neurotransmission, but affects the serotonergic and noradrenergic systems as well (Ritz et al. 1990). Therefore, not only the elevated dopamine levels, but also other cathecholamines might be responsible for non-specific alterations in learning (Breitenstein et al. 2006; Mitchell et al. 2007). In other words, the general pharmacological effects of cocaine may have resulted in more general changes on our task by affecting multiple learning components of the tasks (e.g., saliency or instrumental learning processes), while more specific pharmacological agents like D2 receptor (ant)agonists used in previous studies may have more specific effects.

In contrast to cocaine, cannabis yielded an overall impairment in reversal learning task which was valence-independent. Furthermore, the cannabis-induced impairments were across switch and non-switch trials. We have also found impaired performance on the attention switch task and tower of London. The results also occur with studies cannabis administration studies on other executive functions like response inhibition and error monitoring, which show comparable impairments (Spronk et al. 2015; van Wel et al. 2013; Kowal et al. 2015). Our results suggest that cannabis affects a generic process, rather than exerting any specific effects on cognitive functions. Diminished motivation, or decreased willingness to exert effort, is a well-known side effect of cannabis use (Lynskey and Hall 2000). The results from the current study thus suggest that diminished motivation might underlie impaired performance under acute influence as well.

A number of issues could have influenced the interpretation of our results. First, it is possible that ceiling effects occurred in the cocaine condition. This is of particular relevance for the interpretation of the *DRD2* genotype interaction. The A2/A2 group might, for example, have shown a further cocaine-induced performance increase if the task would have been more difficult. However, the number of participants that reached the maximum score was low, with only a minor difference between the A1 carriers and the A2/A2 group. Second, we used a statistical model (linear-mixed model) that assumes that missing values were missing at random. Most of the missing cases, however, were in the cannabis condition. The extent to which the missing cases were actually random could be questioned, as they were often subjects who were the most tired/least motivated to continue testing. However, we already found an impaired performance under cannabis. If anything, inclusion of those subjects would have demonstrated an even stronger impairment on the level of condition. Third, all subjects reported the use of other substances (most notably XTC, amphetamine, alcohol, and nicotine). The required abstinence, of smoking in particular, could have yielded underperformance in each of the conditions. However, as this effect would have been the same in each of the three conditions, it is unlikely that this had a large effect on the outcomes. Ideally, future studies should address how cocaine and cannabis affect reversal learning in naive users, but ethical concerns limit the feasibility of such studies. Fourth, differences in pharmacodynamics and fatigue effects might have contributed to the dissociative effects on the AST and ToL versus the reversal learning task. The AST and ToL were assessed early in the testing day after the first drug administration, while the reversal learning task was assessed at the very end of the testing day after the second drug administration. Fifth, although our study is the largest of its kind, the power to detect effects of genotype is limited. For the *COMT* gene, genotype data was available for only 59 subjects, with a particularly small Val/Val group (*N* = 13).

In conclusion, this study has demonstrated that acute administration of cannabis and cocaine result in opposing effects on reversal learning and that cocaine’s effects on reversal learning are dependent on individual genetic differences in the dopamine D2 receptor gene.

## Electronic supplementary material

ESM 1 (DOCX 18 kb)
