# Supplementary material for: Acute effects of cocaine and cannabis on reversal learning as a function of COMT and DRD2 genotype
Source: Psychopharmacology (Berl). 2015 Nov 17;233:199–211. doi: 10.1007/s00213-015-4141-5 (PMC4700084; doi:10.1007/s00213-015-4141-5)
Supplement: Supplementary file 1 — (DOCX 18 kb) [file 213_2015_4141_MOESM1_ESM.docx]

# **Supplementary 1**

**Table S-2: Serum concentration (ng.ml^-1^) of THC, THC-COOH, THC-OH and plasma concentrations (ng.ml^-1^) of benzoylecgonine for all four time points. Moment 1 is prior to drug administration, Moment 2 is ~45 min after capsule (T0) and ~ 5 min after vapor administration (T1), Moment 2 is ~10 min after capsule and 5 min after the capsule and vapor booster administration (T2), Moment 4 is at the end of the test day ~ 2 hours after (T2). Results are shown as means±SD. The number of subjects averages were based on is provided in brackets.**

|  | | **M1** | **M2** | **M3** | **M4** |
| --- | --- | --- | --- | --- | --- |
| **Placebo Condition** | |  |  |  |  |
|  | **THC** | 0.55±1.31 (57) | 1.01±1.90 (57) | 0.76±1.21 (57) | 0.52±1.19 (57) |
|  | **THC-OH** | 0.29±0.81 (57) | 0.25±0.65 (57) | 0.20±0.43 (57) | 0.20±0.55 (57) |
|  | **THC-COOH** | 17.64±37.74 (57) | 13.82±27.87 (57) | 12.75±24.18 (57) | 12.54±27.77 (57) |
| **Cannabis Condition** | |  |  |  |  |
|  | **THC** | 1.04±2.67 (60) | 89.09±80.6 (60) | 53.13±42.4 (55) | 4.41±2.86 (54) |
|  | **THC-OH** | 0.31±0.86 (60) | 6.71±3.98 (60) | 5.56±3.55 (55) | 1.97±1.15 (54) |
|  | **THC-COOH** | 15.48±28.3 (60) | 33.81±28.4 (60) | 38.35±35.8 (55) | 28.18±25.0 (54) |
| **Cocaine Condition** | |  |  |  |  |
|  | **THC** | 0.52**±1.24 (58)** | 0.81**±1.63 (57)** | 0.79**±2.11 (58)** | 0.46**±1.19 (58)** |
|  | **THC-OH** | 0.22**±0.56 (58)** | 0.24**±0.60 (57)** | 0.17**±0.42 (58)** | 0.16**±0.40 (58)** |
|  | **THC-COOH** | 19.65**±41.6 (58)** | 18.90**±40.9 (57)** | 14.87**±32.2 (58)** | 13.32**±31.3 (58)** |
|  | **Cocaine** | 0.00±0.00 (57) | 0.22±0.19 (56) | 0.31±0.17 (57) | 0.18±0.094 (57) |
|  | **Benzoylecgonine** | 0.00±0.00 (57) | 0.47±0.30 (56) | 1.20±0.34 (57) | 1.80±0.45 (57) |
